# Diagnostic Performance of a PCR‐Based Approach for the Diagnosis of Dermatomycosis

**DOI:** 10.1111/myc.70127

**Published:** 2025-11-10

**Authors:** Stephan Steixner, Stefan Fuchs, Roya Vahedi‐Shahandashti, Cornelia Lass‐Flörl

**Affiliations:** ^1^ European Excellence Centre of Medical Mycology, Institute of Hygiene and Medical Microbiology Medical University of Innsbruck Innsbruck Austria

**Keywords:** dermatomycosis, dermatophyte diagnosis, dermatophytes, diagnostic workflow, epidemiology, fungal infections, non‐dermatophyte moulds, PCR, *Trichophyton rubrum*

## Abstract

**Background:**

Dermatomycoses, superficial fungal infections of the skin, hair and nails, are among the most common dermatological conditions worldwide. Rapid and accurate diagnosis is essential, particularly in light of emerging antifungal resistance. Conventional diagnostic methods are limited by long turnaround times and lack of species‐level specificity; hence the use of modern DNA‐based tools should be expedient.

**Objectives:**

The aim of this study was to evaluate the diagnostic performance of a pan‐dermatophyte PCR‐based workflow for dermatomycosis in routine diagnostics and to describe the epidemiological landscape in Tyrol, Austria.

**Methods:**

In this retrospective study, 4483 patient specimens (skin, hair and nails) submitted to the Institute of Hygiene and Medical Microbiology, Medical University of Innsbruck, between 2018 and 2024 were analysed. The workflow included initial pan‐dermatophyte PCR, followed by microscopy and fungal culture for PCR‐negative or inconclusive cases. Species identification was performed by Matrix‐assisted Laser‐Desorption Ionisation Time‐of‐Flight mass spectrometry or, if unsuccessful, by sequencing.

**Results:**

Of all specimens, 1170 (26.1%) were PCR‐positive, predominantly with *Trichophyton rubrum* (76.4%), or members of the *T. mentagrophytes‐interdigitale* complex (15.4%). In PCR‐negative but microscopy‐positive samples, 67 dermatophytes were identified by culture. The PCR‐based workflow demonstrated a sensitivity of 94.6%, a negative predictive value of 98.0%, and an overall diagnostic accuracy of 98.5%. Among 335 non‐dermatophyte fungi, *Aspergillus* spp. were most frequent.

**Conclusion:**

The proposed workflow demonstrated high sensitivity and accuracy, supporting its suitability for routine diagnostics. It reduced the need for microscopy and culture while enabling reliable species‐level identification, facilitated epidemiological surveillance, revealing a predominance of *T. rubrum* .

## 1 Introduction

Superficial fungal infections of the skin, hair and nails—collectively termed as dermatomycoses—are among the most common dermatological diseases worldwide and likely represent the most prevalent form of fungal infection, affecting an estimated 20%–25% of the global population [1]. Dermatomycosis is frequently caused by keratolytic fungi called dermatophytes, belonging to the family of Arthrodermataceae. They are grouped into nine genera: *Trichophyton*, *Microsporum*, *Epidermophyton, Arthroderma*, *Nannizzia*, *Ctenomyces*, *Guarromyces*, *Paraphyton* and *Lophophyton*, with *Trichophyton* being the most prevalent [2]. In addition, yeasts such as *Candida* or *Malassezia*, as well as non‐dermatophyte moulds (NDMs) including *Aspergillus*, *Fusarium*, *Acremonium*, or *Scopulariopsis*, can also cause dermatomycosis [3, 4].

Clinical manifestations of dermatomycosis vary considerably by site, skin (tinea), hair (trichomycosis), or nail (onychomycosis), and while usually superficial, they can become invasive in immunocompromised individuals, making accurate diagnosis difficult due to symptom overlap with other conditions [5, 6, 7]. Rapid and precise identification of the causative fungal pathogen is critical to prevent transmission and/or to guide antifungal treatment, especially in cases involving antifungal‐resistant species, e.g., terbinafine‐resistant *T. indotineae* [5, 8, 9, 10]. Accurate diagnosis of dermatomycosis requires species‐level identification, which is often not feasible using conventional methods alone, potentially resulting in misidentification and inappropriate treatment [11, 12, 13, 14]. In addition to the limitations in terms of specificity of conventional diagnostic methods, such as fungal culture, they are also time‐consuming, requiring up to 6 weeks [5, 15]. Nevertheless, they remain valuable, particularly in cases involving atypical skin symptoms, lesions of the scalp or vellus hair regions, suspected NDM or yeast infections, or deep‐seated dermatomycosis [15]. Microscopy, utilising fungal staining, is another conventional diagnostic method that offers a quick alternative and remains essential for avoiding misdiagnosis; however, it also lacks species‐level specificity [15]. To overcome the limitations of conventional methods, newer diagnostic tools have been developed, most notably molecular techniques such as polymerase chain reaction (PCR) and sequencing, which allow for faster and more accurate pathogen identification [11, 15, 16]. PCR offers several advantages over microscopy and fungal culture. It allows direct detection of fungal DNA independent of viability, significantly reduces diagnostic turnaround times from several weeks to just hours, enables species‐level identification when conventional methods such as microscopy or culture fail, and it demonstrates higher sensitivity and specificity compared to conventional techniques, with studies reporting up to 91% sensitivity and reduced false‐negative rates [17, 18, 19, 20]. Moreover, PCR remains effective even in specimens from patients under antifungal treatment, which often impairs culture‐based diagnostic [20, 21]. Given these benefits, updated international guidelines now recommend PCR as complementary or primary diagnostic method in routine dermatological mycology workflows [16, 22].

This retrospective study aimed to evaluate the performance of a diagnostic workflow for the routine diagnostics of dermatomycosis, which is based on a pan‐dermatophyte PCR and complemented by microscopy and fungal culture when necessary, while also providing insights into the current epidemiological landscape in Tyrol, Austria.

## 2 Material and Methods

### 2.1 Sample Collection

In this study we retrospectively analysed skin, hair and nail specimens submitted to the Institute of Hygiene and Medical Microbiology at the Medical University of Innsbruck (HMM‐MUI) from August 2018 to August 2024. Briefly, when the clinician or practitioner had a suspicion for a superficial mycosis, samples were acquired following DIN 58959‐2 standard; sampling was performed either by scalpel scraping (skin and nail), nail clippings for distal nail infections or plucking of hair with epilating forceps [23]. Samples were then submitted promptly by using the MycoTrans Specimen Transport System (MycoTrans Limited, Biggar, UK) by placing the specimens in the MycoTrans envelopes, re‐folding and sealing them for transport to the HMM‐MUI [23].

### 2.2 Routine Diagnostic Workflow

**FIGURE 1:**
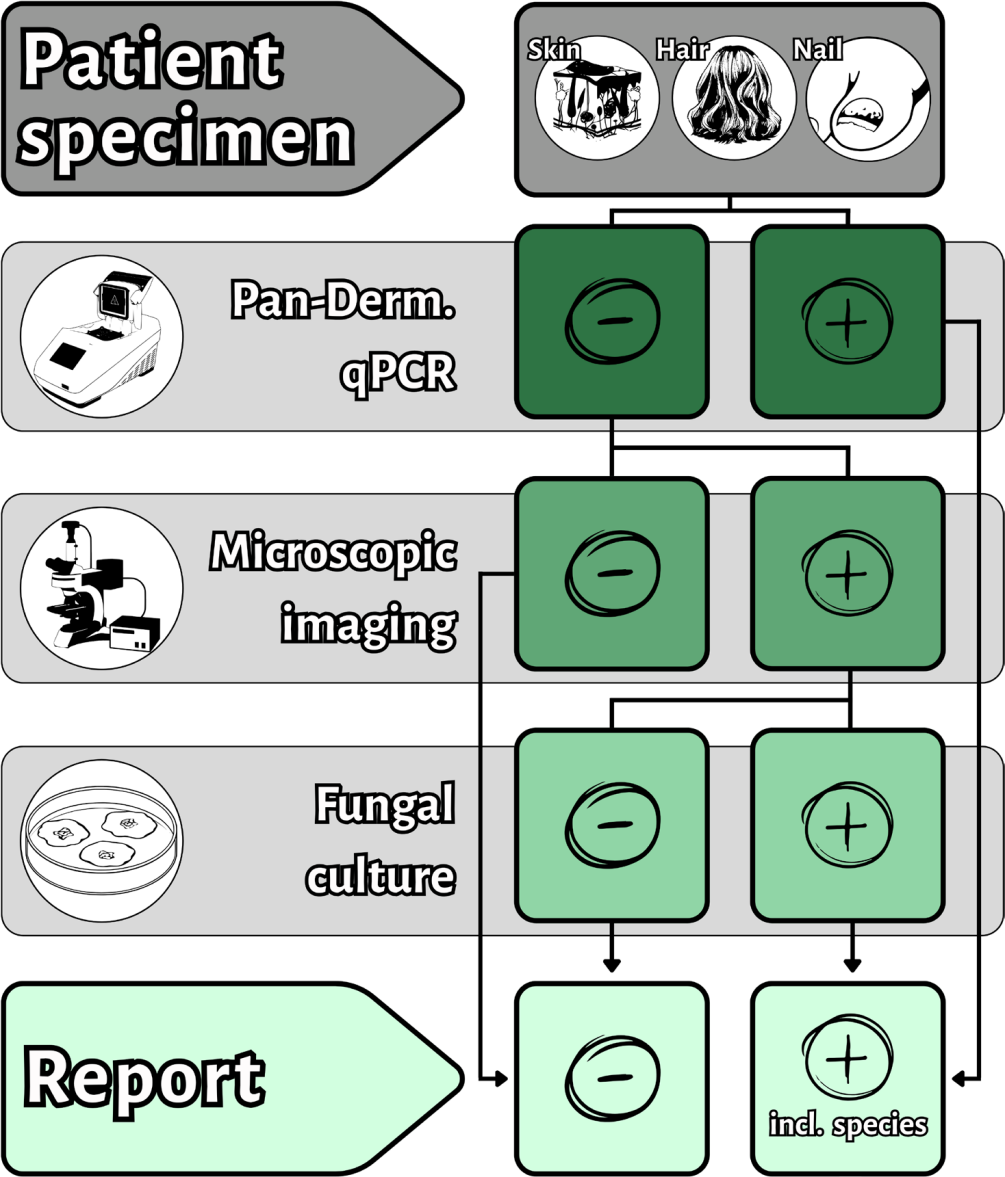
Schematic workflow for the diagnosis of dermatomycosis in skin, hair and nail specimens. The workflow beginning with pan‐dermatophyte PCR, followed by microscopy for PCR‐negative samples, and fungal culture for microscopy‐positive cases. Species identification after positive PCR was performed using multi‐locus sequencing. Culture positive samples were first analysed by Matrix‐assisted Laser‐Desorption Ionisation Time‐of‐Flight mass spectrometry and if inconclusive by multi‐locus sequencing. Incl., including.

The workflow for patient specimens sent to the routine diagnostics laboratory of the HMM‐MUI regarding dermatomycosis diagnostic is illustrated in Figure 1. Patient specimens (skin, hair or nail) are first tested using pan‐dermatophyte qPCR. PCR‐positive samples proceed directly to sequencing for species identification and reporting. PCR‐negative or inconclusive samples undergo calcofluor white (CFW) staining and microscopy. If fungal elements are detected microscopically, culture is performed to retrieve an isolate and identify the species using Matrix‐assisted Laser‐Desorption Ionisation (MALDI) time‐of‐flight (TOF) mass spectrometry. For samples inconclusive in MALDI‐TOF analysis, species is identified by multi‐locus sequencing.

### 2.3 Molecular Diagnostics

In a first step, specimens were prepared for molecular diagnostics using the InnuPREP Forensic Kit (Innuscreen GmbH, Berlin, Germany) in accordance with the manufacturer's instructions. The extracted DNA was then analysed in a validated in‐house pan‐dermatophyte real‐time PCR [24]. In brief, chitin synthase 1 gene (*CHS1*) was amplified, and any generated amplicons were subject to melt‐curve analysis to determine their specificity [17]. Peaks with a melting temperature between 87°C and 92°C and a corresponding amplification curve were considered as positive for dermatophyte DNA. Subsequently, amplicon DNA from PCR‐positive samples was processed using the QIAquick PCR Purification Kit (Qiagen, Hilden, Germany) according to the manufacturer's guide. Sanger sequencing was performed by Eurofins (Eurofins Genomics AT GmbH, Vienna, Austria). Resulting sequences were analysed as described in 2.6.

### 2.4 Microscopic Imaging

For PCR‐negative samples, or PCR‐positive samples with inconclusive sequencing results, microscopy was performed. In summary, depending on the consistency of specimens, parts of the specimens were crushed with a sterile scalpel and denatured with 20% potassium hydroxide (KOH, Merck, Darmstadt, Germany) on a microscopic slide for 1 h. Subsequently, a CFW staining (Fungi‐Fluor Kit, Polysciences Europe GmbH, Hirschberg, Germany) was performed according to the manufacturer's instructions. Stained samples were examined in a fluorescence microscope (Nikon Eclipse E200 with a pE‐300^white^, CoolLED Ltd., Andover, UK) at a magnification of 100× and 400× and investigated for the presence of fungal elements.

### 2.5 Fungal Culture

Fungal culture was only performed on microscopically positive samples with sufficient remaining amounts of specimen. All those samples were inoculated on dermatophyte selective agar (BioMerieux, Marcy l'Etoile, France), Sabouraud Chloramphenicol 2 Agar (BioMerieux, Marcy l'Etoile, France) supplemented with 160 mg/L gentamicin (Serva, Heidelberg, Germany), and liquid culture in Sabouraud Dextrose Broth (HiMedia Laboratories GmbH, Modautal, Germany) supplemented with 80 mg/L chloramphenicol (Sigma‐Aldrich, Vienna, Austria). Samples were incubated at 28°C for a maximum period of 6 weeks. For skin specimens, in addition to the above‐mentioned culture media, a Sabouraud Chloramphenicol 2 Agar plate was inoculated with the specimen and overlaid with olive oil to enhance the growth of *Malassezia* spp. [25]. Daily controls of growth were conducted.

### 2.6 Species Identification

Fungal species identification from positive fungal culture was routinely performed by MALDI‐TOF mass spectrometry using a MALDI Biotyper smart (Bruker Daltonics, Bremen, Germany). For the species identification we used the Mycelium Transfer procedure described by the manufacturer. In short, small portions of fungal colonies were applied to a MBT Biotarget 96 IVD (Bruker Daltonics, Bremen, Germany). Samples were covered with 1 μL of 70% formic acid and dried. Thereafter, samples were covered with 1 μL of matrix solution (α‐cyano‐4‐hydroxycinnamic acid) and dried. The target was finally inserted in the MALDI‐TOF mass spectrometer for analysis. MALDI‐TOF mass spectrometry data was compared to the “filamentous fungi” database from Bruker (Bruker Daltonics, Bremen, Germany). As specified by the manufacturer, scores of ≥ 2.0 were considered sufficient for actual identification. Morphological characteristics of the fungal growth were also analysed, using a lactophenol blue staining (Merck, Darmstadt, Germany). For cases, in which species identification by morphology and MALDI‐TOF was unsuccessful, a multi‐locus amplification‐sequencing approach was used to identify the species of the isolate [26]. Therefore, amplicons were Sanger‐sequenced by Eurofins (Eurofins Genomics AT GmbH, Vienna, Austria) and the final identification of species was done using BLAST search and a pairwise alignment (Mycobank, https://www.mycobank.org/page/Pairwise_alignment). Species results were submitted as the final microbiological report to the clinician or practitioner.

### 2.7 Statistical Analysis

Trends in annual specimen submission were analysed using linear trend analysis. The drop in sample number was evaluated using a Z‐score analysis based on the mean and standard deviation of the yearly sample number. Diagnostic parameters such as sensitivity, negative predictive value and diagnostic accuracy were calculated according to the literature [27, 28].

### 2.8 Ethics

The authors confirm that the ethical policies of the journal, as noted on the journal's author guidelines page, have been adhered to. As all samples were fully anonymised before analysis and no patient data were recorded, no ethical approval or informed consent were required.

## 3 Results

In the 6‐year study period (August 2018 to August 2024), a total of 4483 patient specimens were dispatched to the HMM‐MUI. Over the study period, the number of specimens submitted for dermatophyte diagnostics increased significantly from 375 samples in 2018 to 806 in 2024 (*p* = 0.037). During the early phase of the COVID pandemic in 2020, a substantial decrease to 328 specimens was observed (*z*‐score = −2.06). The 4483 patient specimens comprised 1680 skin (37.5%), 75 hair (1.7%), and 2728 nail specimens (60.8%). Utilising our comprehensive PCR‐based approach, we successfully identified 1237 dermatophytes in all patient samples. Additionally, 335 non‐dermatophyte fungi, including moulds (73.1%) and yeasts (26.9%), were identified by fungal culture. Out of the 1237 dermatophytes identified, *Trichophyton* spp. (96.2%) were by far the most prevalent, followed by *Microsporum* spp. (1.8%), *Nannizzia* spp. (1.2%) *Arthroderma* spp. (0.6%) and *Epidermophyton* spp. (0.2%). Within the genus of *Trichophyton* (*n* = 1190), *T. rubrum* was the most frequent species (79.3%), followed by the *T. mentagrophytes‐interdigitale* complex (15.5%) and other *Trichophyton* spp. (5.2%) such as *T. tonsurans* , *T. benhamiae* , *T. uncinatum* , *T. verrucosum* and *T. violaceum* . With our PCR‐based approach, a sensitivity of 94.6% and a negative predictive value of 98.0% were achieved, resulting in an overall accuracy of 98.5% and a false‐negative rate of 5.4% regarding the PCR.

### 3.1 PCR‐Positive Samples

**FIGURE 2:**
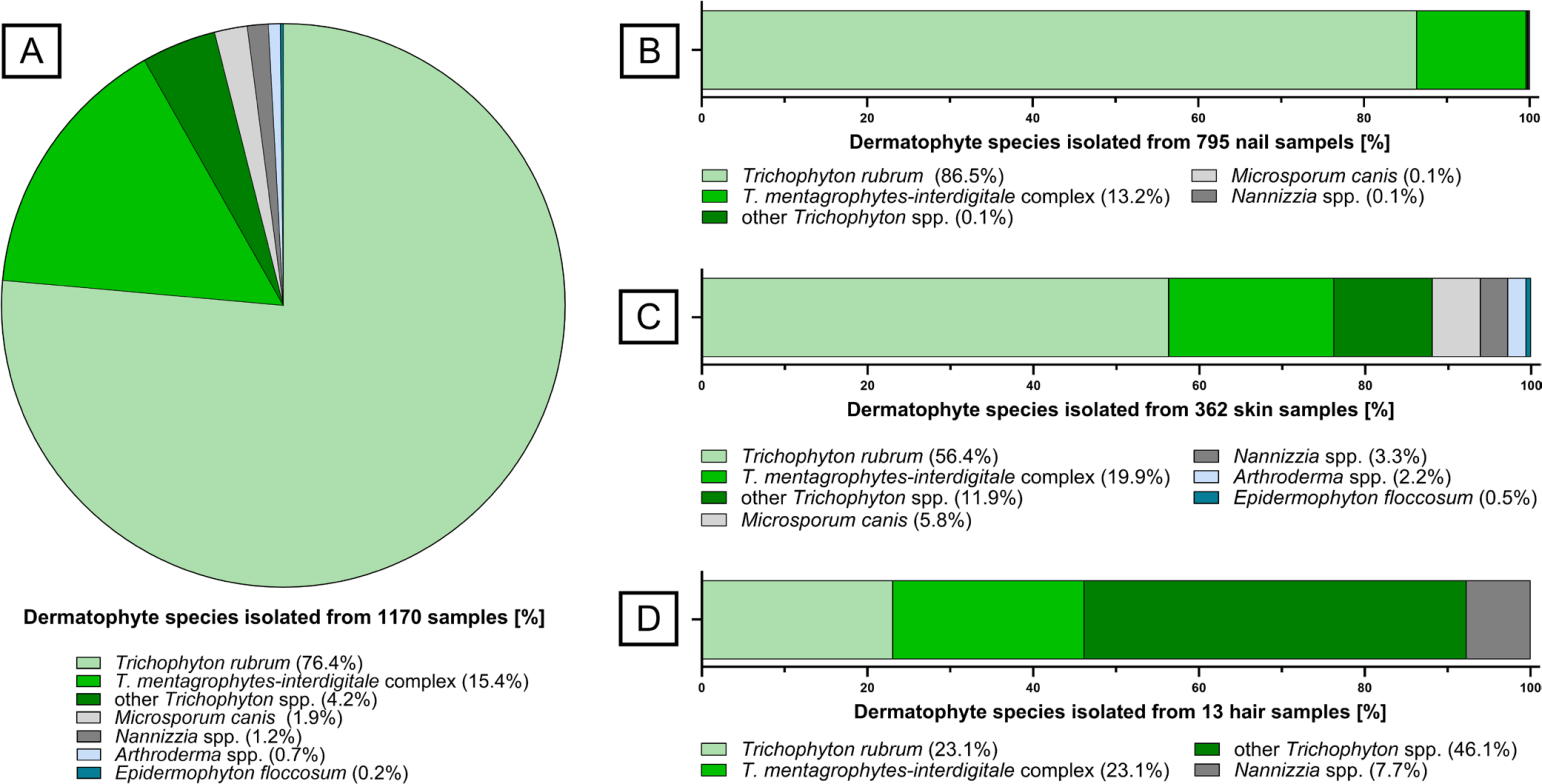
Distribution of dermatophyte species of 1170 PCR‐positive patient specimens. (A) All 1170 specimens, (B) 795 nail specimens, (C) 362 skin specimens, (D) 13 hair specimens.

Of the 4483 analysed samples, 1170 (26.1%) exhibited a positive PCR result. Subsequent species identification by *CHS1* sequencing is summarised in Figure 2. In all 1170 PCR‐positive samples, including 362 skin, 13 hair and 795 nail specimens (Figure 2A), *T. rubrum* (76.4%) was the predominant species followed by the *T. mentagrophytes‐interdigitale* complex (15.4%), other *Trichophyton* spp. (4.2%) such as *T. tonsurans, T. benhamiae, T. uncinatum, T. verrucosum* and *T. violaceum* , *M. canis* (1.9%), *Nannizzia* spp. (1.2%), *Arthroderma* spp. (0.7%) and *E. floccosum* (0.2%). Upon further classification into specimen types (Figure 2B–D), *T. rubrum* remained the predominant pathogen in skin (56.4%) and nail (86.5%) specimens, with the exception of hair specimens, where *T. benhamiae* (*n* = 1) and *T. verrucosum* (*n* = 5) were found to be more prevalent (46.1%). *Arthroderma* spp. and *E. floccosum* were only detected in low quantities in skin specimens.

### 3.2 PCR‐Negative Samples

**FIGURE 3:**
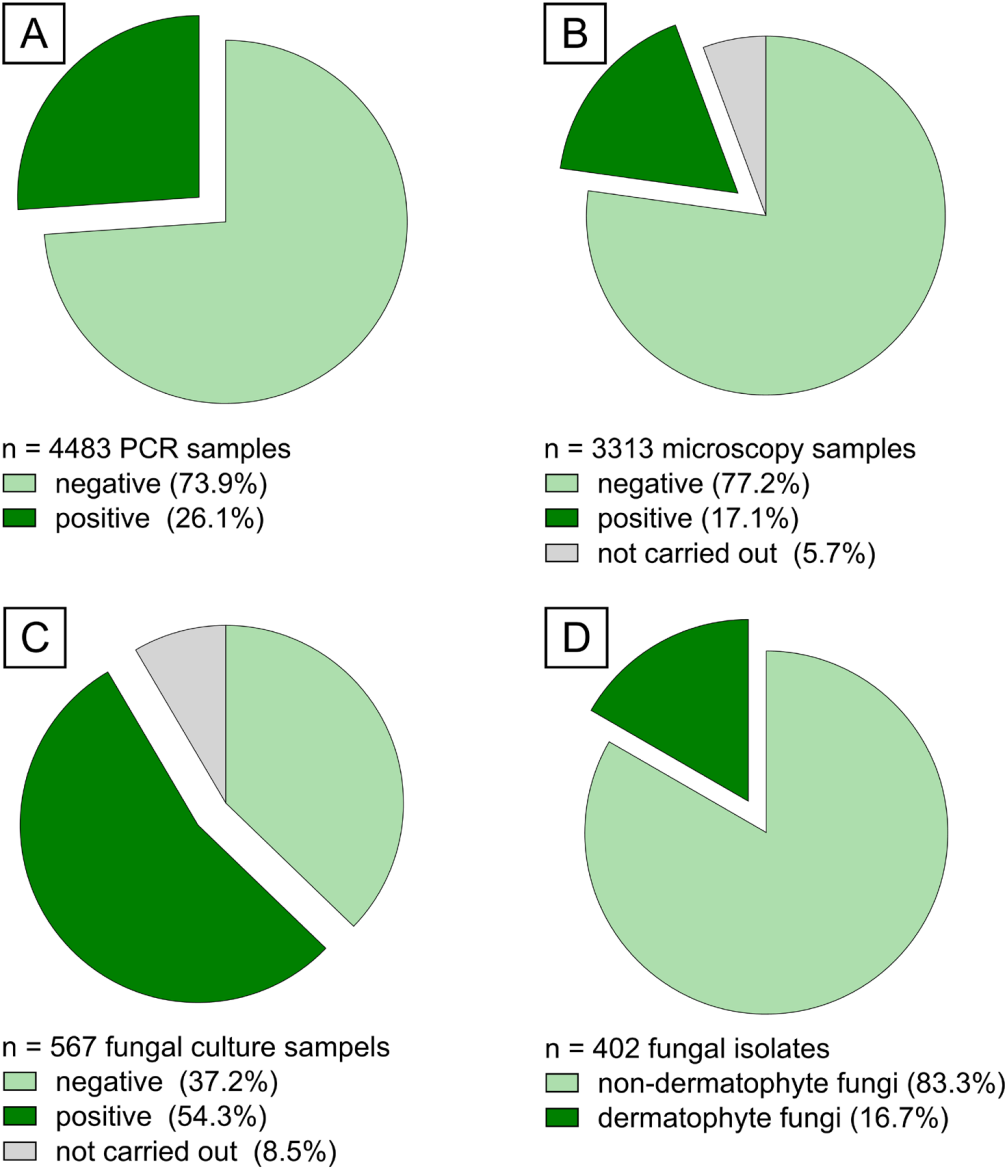
Diagnostic outcomes of 4483 patient specimens analysed using the stepwise workflow (section 2.2). (A) Initial testing of all 4483 samples with pan‐dermatophyte PCR, (B) 3313 PCR‐negative specimens examined by calcofluor white (CFW) staining, (C) 567 CFW‐positive samples subsequently cultured for fungal identification, (D) 402 fungal isolates of 308 culture‐positive samples.

After primary analysis with the dermatophyte‐specific PCR, 3313 samples (73.9%) were negative for dermatophyte DNA (Figure 3). In the subsequent step, 188 samples had to be excluded from further analysis due to insufficient amounts of specimen remaining for microscopy. The remaining 3125 samples were subject to analysis by CFW staining. Of all microscopic samples, 2558 (81.9%) showed no fungal presence in microscopy and were directly reported as negative for dermatomycosis. All samples that contained fungal elements in microscopy were subsequently subjected to culturing, as outlined in section 2.5. Some samples (*n* = 48) had to be excluded from further processing due to insufficient amounts of material, resulting in a total of 519 samples for culture. Of the remaining samples, 211 (40.7%) samples did not show any fungal growth after 6 weeks and were thus reported as negative for dermatomycosis.

**FIGURE 4:**
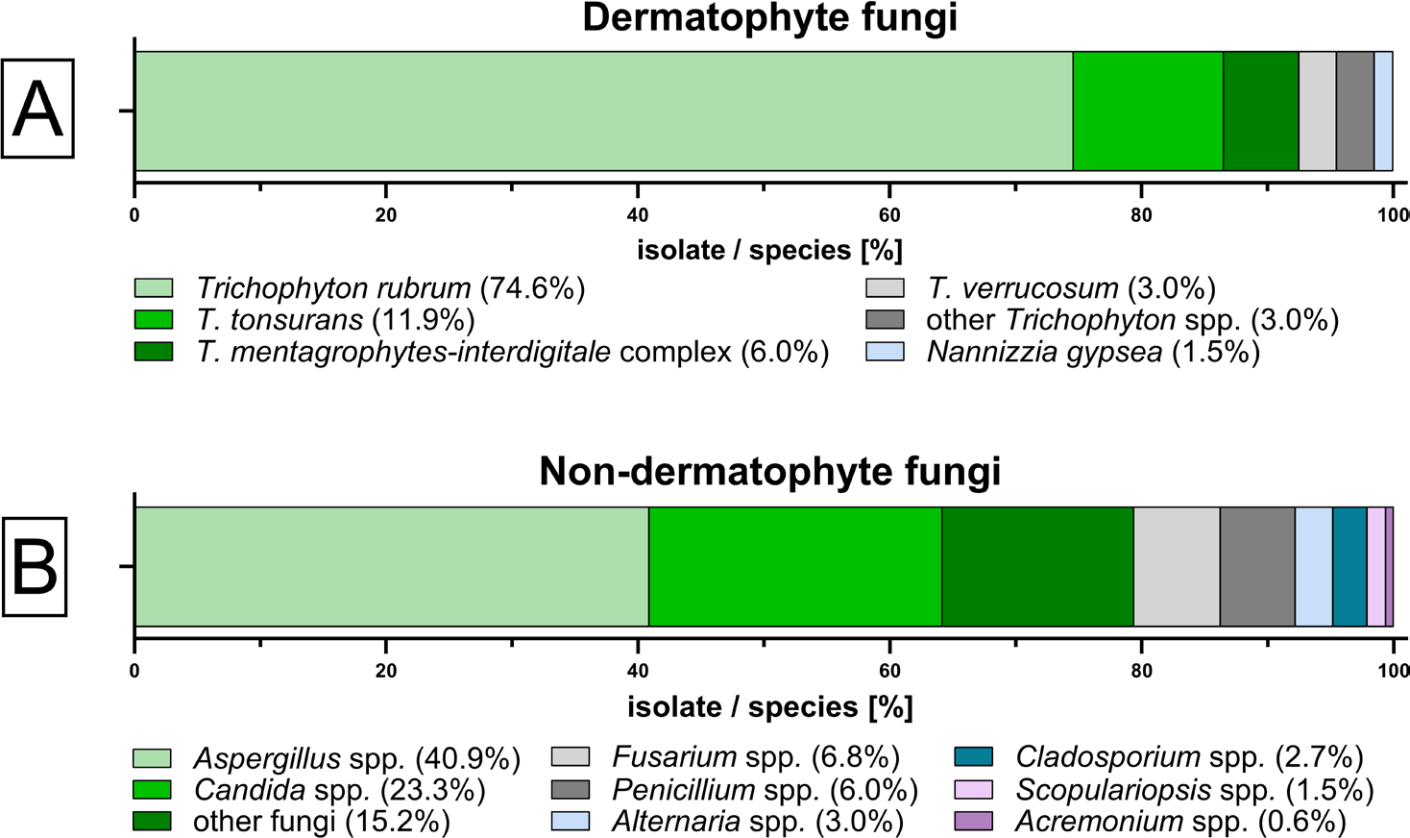
Species distribution of fungal isolates identified by culture from PCR‐negative samples with microscopy‐proven fungal elements. Bar plots show the proportions of (A) 67 dermatophyte isolates and (B) 335 non‐dermatophyte fungal isolates recovered from 308 patient specimens with negative PCR results but positive in microscopy and culture.

The results of fungal culture are summarised in Figure 4. A total of 308 samples (6.9%) of all 4483 samples between August 2018 and August 2024 showed negative PCR results but positive microscopy and fungal culture. Some samples showed growth of multiple fungal species; therefore 402 fungal isolates were successfully cultured. Out of all samples positive in culture, 67 (21.8%) were identified as dermatophytes with *T. rubrum* (74.6%) as the leading species, followed by *T. tonsurans* (11.9%), *T. mentagrophytes‐interdigitale* complex (6.0%), *T. verrucosum* (3.0%), other *Trichophyton* spp. (3.0%) and *N. gypsea* (1.5%). The remaining 335 fungi were identified as belonging to various other species, including NDMs, such as *Aspergillus* spp. (40.9%), or *Candida* spp. (23.3%).

## 4 Discussion

Effective management of superficial fungal infections requires accurate identification of the causative agent, as many dermatological conditions can mimic tinea infections. Conventional methods such as culture and microscopy, although still considered gold standards, are limited by low sensitivity, lack of species‐level specificity and long turnaround times [21, 29]. However, in view of these limitations, molecular diagnostic techniques, particularly PCR‐based approaches, are increasingly adopted for their higher sensitivity, specificity and rapid results [11, 16, 18, 20]. Studies have shown that the highest diagnostic accuracy can be achieved by combining conventional and molecular techniques, and the 2023 S1 guideline further recommends PCR as a key adjunct to traditional methods [15, 16]. Incorporation of molecular approaches into routine dermatomycosis diagnostics might help to overcome the limitations of the classical approaches.

In this study, we retrospectively evaluated the diagnostic performance of a PCR‐based workflow for dermatomycosis detection in routine diagnostics. The workflow, starting with a pan‐dermatophyte PCR, followed by microscopy for PCR‐negative or inconclusive samples, and fungal culture on microscopy‐positive cases, was empirically applied to optimise diagnostic yield, reduce turnaround time and conserve resources. In the traditional diagnostic workflow, diagnosis typically begins with microscopy, followed by culture, with PCR often included only as a supplementary test [15, 21]. In contrast, the workflow applied here starts with PCR, enabling rapid and species‐level detection of dermatophytes within a few days, which is particularly advantageous in clinical settings where timely treatment decisions are critical [16, 19, 20]. In our workflow we used a pan‐dermatophyte qPCR with subsequent multi‐locus sequencing for species identification. In contrast to commercial PCR kits, our combination of qPCR and sequencing enables detection of a broader range than commercial qPCR kits, which show good species coverage, but still lack some. However, the cost‐effectiveness, laboratory workload and turnaround time of the applied workflow were not systematically evaluated, which limits our ability to draw conclusions about its broader implementation. Sequencing of PCR‐positive samples provides species‐level identification and supports epidemiological analysis; however, it requires technical resources and might not be feasible in all routine laboratories. By limiting microscopy and culture to PCR‐negative samples, the burden of microscopy was effectively reduced by 26.1% and of culture by 87.4%, while still ensuring diagnostic coverage for cases involving NDMs, yeasts, or PCR lapses. While simultaneous microscopy and culture may improve diagnostic yield, both methods have well‐documented limitations [15, 22]; therefore, this study focuses on the evaluation of the diagnostic workflow algorithm instead.

In our study, among the 1237 dermatophyte‐positive cases identified, PCR alone failed to detect 67 cases (5.4%), which were subsequently confirmed through microscopy and culture. The discrepancies between the PCR results and the fungal culture might reflect DNA degradation, low pathogen load equalling insufficient DNA, sample inhomogeneities, or primer mismatch in the case of genetically divergent strains [18, 20, 30]. False‐negative PCR results may delay treatment initiation; thus, integrating clinical suspicion and complementary diagnostics remains essential [16, 18, 20]. The PCR‐based approach in this study achieved a sensitivity of 94.6%, exceeding clearly the 82%–91% reported in previous studies [18, 19], with a negative predictive value of 98.0% and an overall accuracy of 98.5%, highlighting the robustness of the outlined workflow. PCR commonly allows direct species identification, even when microscopy or culture fails; however, a key limitation is its inability to distinguish viable from non‐viable organisms, requiring careful clinical interpretation [11, 18]. Due to the retrospective design of our study, not all specimens underwent all three diagnostic methods, which limits a direct comparison of their performance. Nevertheless, the stage‐wise strategy applied here may still represent a pragmatic and resource‐efficient approach, balancing speed, diagnostic accuracy and cost, and aligning with recent recommendations for incorporating molecular methods into routine dermatomycosis diagnostics.

This diagnostic workflow not only demonstrated strong diagnostic performance but also provided valuable epidemiological insights. Across the entire diagnostic algorithm, *Trichophyton* spp. were the most frequently identified dermatophytes, with *T. rubrum* (96.2%) being predominant. This contrasts with findings from Southern and Eastern Europe, where the *T. mentagrophytes‐interdigitale* complex remains more prevalent. It highlights the dominance of *T. rubrum* in Central Europe [31]. However, in hair samples, species like *T. verrucosum* and *T. benhamiae* were more frequent confirming recent observations on their epidemiology [32]. Furthermore, contrary to the literature, we did not detect *M. canis* in our hair specimens [33, 34, 35]. However, with only 75 hair specimens, we received a substantially lesser amount of samples compared to other specimen types, which could be explained by the relatively low prevalence of trichomycosis of 3%–4% of all dermatomycosis cases reported in several European countries [32, 36]. The emergence of *T. indotineae* (formerly designated as the *T. mentagrophytes‐interdigitale* complex genotype VIII), a terbinafine‐resistant lineage spreading from India, has already been detected in Europe, including Austria, and poses a growing medical concern due to its resistance characteristics and pronounced virulence [12, 13, 14, 37, 38]. Accurate identification of T. *indotineae* requires further molecular identification, such as specific PCRs, internal transcribed spacer (ITS) sequencing or MALDI‐TOF mass spectrometry [39, 40, 41, 42, 43, 44, 45]. Differentiation for *T. indotineae* by ITS sequencing from fungal culture was only implemented in 2023; therefore, not all samples were screened for *T. indotineae*. In the remainder of the study period, no *T. indotineae* isolates were identified; however, two *T. indotineae* cases have been confirmed from culture material since the end of the study period. As our study did not include clinical patient data, possible explanations and influences of age, gender, or other patient‐related factors could not be analysed. Especially, the age of patients has shown to be influential not only in the type of dermatomycosis, but also in the etiological agent of infection [46].

While dermatophytes remain the main cause of dermatomycosis, NDMs are increasingly reported to account for approximately 6.9% of cases globally (range 1.8%–23.5%), with varying prevalence in Europe ranging from 4.1% to 11.5% [47, 48, 49, 50, 51, 52, 53, 54, 55, 56]. Worldwide, *Aspergillus* spp. (45.3%), *Scopulariopsis* spp. (13.2%) and *Fusarium* spp. (11.6%) are the most common NDMs, while in Europe, *Scopulariopsis* spp. is more prevalent (29.3%) [53]. In contrast to the reported European trend of *Scopulariopsis* dominance, we observed a predominance of *Aspergillus* spp. among NDMs. However, as our real‐time PCR targets only dermatophytes, data on NDMs and yeasts were only derived from PCR‐negative samples with positive fungal culture. The clinical relevance of NDMs remains debated. While dermatophyte detection strongly indicates infection, NDMs also may represent colonisation, contamination, or secondary infection [53]. Nonetheless, some NDMs, like *Aspergillus* spp. or *Fusarium* spp., show keratolytic activity in vitro, supporting their potential pathogenic role [57, 58].

## 5 Conclusion

This retrospective analysis shows that, by prioritising pan‐dermatophyte PCR as the initial step in routine dermatomycosis diagnostics and utilising microscopy and culture only for PCR‐negative or inconclusive samples, we achieved a considerably high diagnostic accuracy and sensitivity, while markedly reducing workload and turnaround time. This approach not only improves pathogen detection but also enables species‐level identification at the time of diagnosis and provides valuable epidemiological insights. Despite its retrospective design and the absence of a cost‐effectiveness analysis, our findings corroborate the integration of molecular methods, particularly the pan‐dermatophyte PCR, as a cornerstone in routine dermatological mycology workflows, in line with current international recommendations. Prospective studies assessing cost‐effectiveness and correlations with clinical outcomes are warranted to further validate this strategy.

## Author Contributions

**Stephan Steixner:** conceptualization, methodology, visualization, validation, writing – original draft, writing – review and editing, investigation, formal analysis, data curation. **Stefan Fuchs:** writing – review and editing, visualization, conceptualization, investigation. **Roya Vahedi‐Shahandashti:** conceptualization, writing – original draft, writing – review and editing, visualization. **Cornelia Lass‐Flörl:** supervision, resources, project administration, writing – review and editing, conceptualization, funding acquisition, investigation.

## Conflicts of Interest

The authors declare no conflicts of interest.

## Data Availability

All data is provided within the article.
